# Control Group Design, Contamination and Drop-Out in Exercise Oncology Trials: A Systematic Review

**DOI:** 10.1371/journal.pone.0120996

**Published:** 2015-03-27

**Authors:** Charlotte N. Steins Bisschop, Kerry S. Courneya, Miranda J. Velthuis, Evelyn M. Monninkhof, Lee W. Jones, Christine Friedenreich, Elsken van der Wall, Petra H. M. Peeters, Anne M. May

**Affiliations:** 1 Julius Center for Health Sciences and Primary Care, University Medical Center, Utrecht, the Netherlands; 2 Faculty of Physical Education and Recreation, University of Alberta, Edmonton, Canada; 3 Comprehensive Cancer Center the Netherlands (IKNL), Utrecht, the Netherlands; 4 Memorial Sloan-Kettering Cancer Center, New York, New York, United States of America; 5 Department of Population Health Research, Cancer Control Alberta, Alberta Health Services, Calgary, Canada; 6 Department of Community Health Sciences, Faculty of Medicine, University of Calgary, Calgary, Canada; 7 Department of Oncology, Faculty of Medicine, University of Calgary, Calgary, Canada; 8 Cancer Center, University Medical Center Utrecht, Utrecht, the Netherlands; Johns Hopkins Bloomberg School of Public Health, UNITED STATES

## Abstract

**Purpose:**

Important considerations for exercise trials in cancer patients are contamination and differential drop-out among the control group members that might jeopardize the internal validity. This systematic review provides an overview of different control groups design characteristics of exercise-oncology trials and explores the association with contamination and drop-out rates.

**Methods:**

Randomized controlled exercise-oncology trials from two Cochrane reviews were included. Additionally, a computer-aided search using Medline (Pubmed), Embase and CINAHL was conducted after completion date of the Cochrane reviews. Eligible studies were classified according to three control group design characteristics: the exercise instruction given to controls before start of the study (exercise allowed or not); and the intervention the control group was offered during (any (e.g., education sessions or telephone contacts) or none) or after (any (e.g., cross-over or exercise instruction) or none) the intervention period. Contamination (yes or no) and excess drop-out rates (i.e., drop-out rate of the control group minus the drop-out rate exercise group) were described according to the three design characteristics of the control group and according to the combinations of these three characteristics; so we additionally made subgroups based on combinations of type and timing of instructions received.

**Results:**

40 exercise-oncology trials were included based on pre-specified eligibility criteria. The lowest contamination (7.1% of studies) and low drop-out rates (excess drop-out rate -4.7±9.2) were found in control groups offered an intervention after the intervention period. When control groups were offered an intervention both during and after the intervention period, contamination (0%) and excess drop-out rates (-10.0±12.8%) were even lower.

**Conclusions:**

Control groups receiving an intervention during and after the study intervention period have lower contamination and drop-out rates. The present findings can be considered when designing future exercise-oncology trials.

## Introduction

There is growing evidence for beneficial effects of physical exercise in patients with cancer [1–3]. Pooled results of exercise oncology trials focusing on supervised and home-based exercise programs in several meta-analyses showed that exercise during and after cancer treatment is beneficial in terms of improved quality of life, physical fitness, reduced cancer-related fatigue and anxiety and depression [1–3]. Reported effect sizes were small to moderate, which might partially be explained by possible contamination in the control groups (i.e. control participants adopt the exercise intervention) [4].

In many exercise-oncology trials, patients are randomized to either an exercise intervention or a usual care (i.e., no exercise) control group. Given the nature of the intervention, blinding of the participants to their allocation is not possible, which then can create undesirable consequences in these trials because of the control group. First, inclusion of participants for the trial may be affected because eligible patients decide to refrain from participation because they do not want to be randomized to the control group [5]. Second, cancer patients participating in an exercise trial are highly motivated to exercise and, therefore, participants randomized to the control group may also increase their physical activity levels. They often change their behavior despite the request to maintain their usual activity pattern [6]. This non-compliance by controls may lead to a decrease of power to detect a significant intervention effect. Last, patients who first agreed to participate may drop out after being randomized to the control group.

In exercise trials, different designs have been used to address these issues with the control group participants in terms of: (A) the exercise instruction before the start of the intervention, (B) the intervention offered to controls during the study intervention and (C) the intervention offered to controls after the intervention [7]. In some trials the control group is asked to either maintain their usual lifestyle pattern during the study period or to refrain from exercise. Other trials offered the control group an alternative intervention during the study period, like stretching exercises. Finally, in some studies an intervention is offered after completion of the study. Thus far, the influence of these different study designs on contamination and drop-out within the controls has not been studied.

This review provides an overview of these different types of control groups applied in exercise-oncology trials and explores the influence on contamination and drop-out rates. In addition, we will explore the impact of restriction of pre-trial exercise level on contamination and drop-out rates. The aim of this review is to provide useful information to improve the design of future exercise oncology trials.

## Methods

### Search strategy

We included exercise oncology trials from two relevant Cochrane reviews on the effect of exercise during and after cancer treatment on fatigue and quality of life [1, 3]. Furthermore, we performed a computer-aided search for the trials published after these reviews (April 19, 2012 to August 16, 2013) using Medline (Pubmed), Embase and CINAHL. The following search terms were used: ‘exercise (with synonyms) and ‘cancer’ (with synonyms) and ‘trials’ (see S1 Table). The reference lists of identified studies were searched for additional relevant studies.

### Inclusion criteria

We included randomized controlled trials investigating the effect of supervised or home-based exercise training on quality of life or cancer-related fatigue performed in adults older than 18 years diagnosed with cancer and treated curatively. Included studies evaluated the effects of exercise programs offered before, during or after their cancer treatment. Excluded were trials primarily aimed at behavioral change. We also excluded small pilot studies (<20 patients per study arm). Small studies were excluded because a small sample size may affect our study outcome: less effort is needed to prevent high drop-out rates or low study compliance than in large trials. No language restrictions were used.

### Selection of studies

Two independent reviewers (CS, AM) screened the titles and abstracts of all identified studies for eligibility (n = 159). Full texts of potential relevant papers were subsequently read for inclusion.

### Data extraction

For each study the following data were extracted: patient and disease characteristics (age and cancer type), information about the exercise intervention (i.e. timing of exercise intervention according to treatment; aerobic or resistance training; and supervised or home-based exercise, restriction of pre-trial exercise level (none or inactive/unfit), excess drop-out rates and contamination.

We computed the excess drop-out rate for each study by subtracting the drop-out rate in the exercise group from the drop-out rate in the control group. This estimation was done to standardize drop-out rates across studies.

We rated contamination (yes or no) for each study as defined by Waters et al. (2012) [7] as an increase of ≥ 60 minutes (= 4 Metabolic Equivalent of Task (MET)-hours = 1 kcal/kg/hour) of moderate to vigorous physical activity per week in the control group, or a 10% increase in the proportion of participants in the control group meeting the study exercise prescription (e.g. physical activity guidelines). In addition, contamination was also scored to be present if reported by the authors using slightly different definitions. For example, in several home-based exercise trials, contamination was defined as exercising (moderate/strenuous) >60 minutes per week [8].

Two independent reviewers (CS, AM) extracted all information for all trials.

### Data analysis

Data were described per study and according to three design characteristics of the control group:

- the exercise instruction given to the controls before start of the study: exercise allowed (n = 18) or exercise not allowed (n = 22).
- the intervention the control group was offered during the intervention period: any intervention offered (n = 21) or no intervention offered (n = 19). Any intervention included information about exercise (n = 1), education sessions (n = 4), regular telephone contacts (n = 6), stretching sessions (n = 2), relaxation training (n = 1), exercise diary (n = 2), regular phone calls and use of pedometers (n = 1) or regular phone calls and use of exercise diary or pedometers (n = 4). Note, that because of small numbers of studies these different interventions were combined into one category.
- the intervention for the control group was offered after the intervention period had ended: any intervention offered (n = 19) or no intervention offered (n = 21). Any intervention included a full cross-over study (n = 7), a partial cross-over (n = 2) or exercise prescriptions (n = 10).
  Each study was grouped according to the three design characteristics of the control group (A, B, C) and according to the combinations of these three characteristics. Data per subgroup were shown descriptively. Mean and standard deviations of the drop-out rates and contamination rates were calculated. Mean excess drop-out rates and contamination were also described by pre-trial exercise level (no restriction or inactive/unfit). Unfortunately, because of limited numbers performing inferential statistical analyses to test differences between groups and/or controlling for confounding factors was not feasible.

## Results

**Fig 1:**
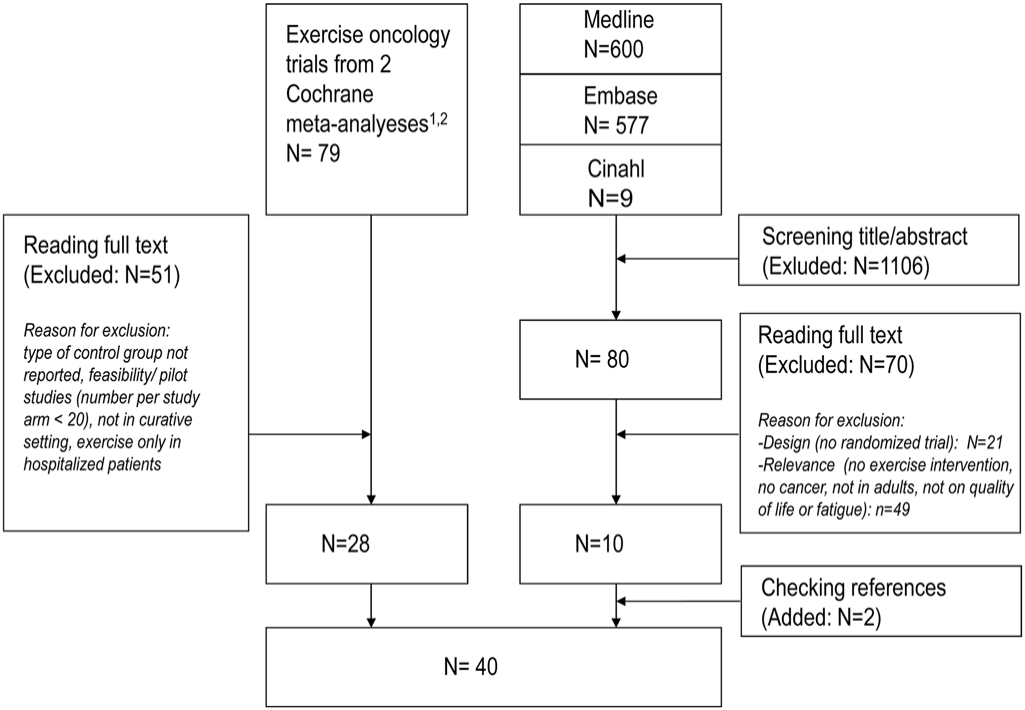
Flowchart of included studies. Exercise oncology trials of the literature search between 19-4-2012 and 16-8-2013 and from two Cochrane reviews updated on 19-4-2012 and 1-6-2012. [1,2].

From the two Cochrane reviews [1, 3] we included 28 studies. In the additional literature search, 1186 studies were found, of which 10 studies met the inclusion criteria. Checking references in the papers led to two additional references. Fig. 1 shows the flowchart of included studies [4, 8–42]. Characteristics of each individual study are described in S2 Table.

Overall, information on contamination was reported in 30 out of 40 studies (75%) and drop-out rates were provided in 36 out of 40 studies (90%). Most studies included patients with mixed cancer types (40%), or with breast cancer only (38%). Most interventions were performed after treatment (35%), were supervised (48%),included an aerobic (55%) exercise program and included participants without pre-trial exercise restrictions (70%) (Table 1).

**Table 1:** Summary of characteristics of included studies.

	All studies	A.Instruction control group BEFORE study intervention		B.Intervention control group DURING study intervention		C.Intervention control group AFTER study intervention	
		Exercise allowed^a^	No change^b^	Anything^c^	None	Anything^d^	None
Total number of studies	40	22	18	21	19	19	21
Study size							
< 60	10 (25%)	6 (27%)	4 (22%)	3 (14%)	7 (37%)	6 (32%)	4 (19%)
≥ 60	30 (75%)	16 (73%)	14 (78%)	18 (86%)	12 (63%)	13 (68%)	17 (81%)
Mean age (years)							
<60	32 (80%)	17 (77%)	15 (83%)	16 (76%)	16 (84%)	14 (74%)	18 (86%)
≥ 60	8 (20%)	5 (23%)	3 (17%)	5 (24%)	3 (16%)	5 (26%)	3 (4%)
Restriction of pre-trial exercise level							
None	28 (70%)	18 (82%)	10 (56%)	12 (57%)	16 (84%)	13 (68%)	15 (71%)
Inactive	12 (30%)	4 (18%)	8 (44%)	9 (43%)	3 (16%)	6 (32%)	6 (29%)
Cancer type							
Breast	16 (40%)	8 (36%)	8 (44%)	7 (33%)	9 (47%)	7 (37%)	9 (43%)
Lung	1 (3%)	1 (5%)	-	1 (5%)	-	-	1 (5%)
Lymphoma	1 (3%)	-	1 (6%)	-	1 (5%)	1 (5%)	-
Prostate	5 (13%)	4 (18%)	1 (6%)	2 (10%)	3 (16%)	4 (21%)	1 (5%)
Colorectal	2 (5%)	1 (5%)	1 (6%)	2 (10%)	-	1 (5%)	1 (5%)
Head&Neck	2 (5%)	-	2 (11%)	1 (5%)	1 (5%)	1 (5%)	1 (5%)
Mixed	13 (33%)	8 (36%)	5 (28%)	8 (38%)	5 (26%)	5 (32%)	8 (38%)
Timing study intervention according to treatment							
During chemo	7 (18%)	4 (18%)	3 (17%)	5 (24%)	2 (11%)	3 (16%)	4 (19%)
During + after chemo	6 (15%)	4 (18%)	2 (11%)	3 (14%)	3 (16%)	4 (21%)	2 (10%)
After treatment	14 (35%)	6 (27%)	8 (44%)	7 (33%)	7 (37%)	6 (32%)	8 (38%)
Mixed/ Other	13 (33%)	8 (36%)	5 (28%)	6 (29%)	7 (37%)	6 (32%)	7 (33%)
Type of exercise							
Aerobic	22 (55%)	8 (36%)	14 (78%)	12 (57%)	10 (53%)	11 (58%)	11 (52%)
Resistance	2 (5%)	2 (9%)	-	1 (5%)	1 (5%)	1 (5%)	1 (5%)
Combination	16 (40%)	12 (55%)	4 (22%)	8 (38%)	8 (42%)	7 (37%)	9 (43%)
Setting of exercise intervention							
Supervised	19 (48%)	12 (55%)	7 (39%)	6 (29%)	13 (68%)	11 (58%)	8 (38%)
Home based	14 (35%)	5 (23%)	9 (50%)	11 (52%)	3 (1650	7 (37%)	7 (33%)
Combination	7 (18%)	5 (23%)	2 (11%)	4 (19%)	3 (16%)	1 (5%)	6 (29%)

Data are given in number of studies and percentages (N (%)).

Chemo = chemotherapy

^a^. Instruction for control group included: exercise allowed during the intervention period, including no advice, asked or recommended to exercise.

^b^. Instruction for control group included: not to exercise, no change in exercise, continue exercise as it is.

^c^. Intervention for control group included: information about exercise, education (session) unrelated to exercises, keep exercise diary, use pedometers or accelerometers, phone calls unrelated to exercise, alternative intervention.

^d^. Study intervention was (partially) offered to control group after study intervention period, or information about exercise or exercise prescriptions were offered to control group after study intervention period.

On average the studies had lower drop-out rates in the control as compared to the exercise intervention group (-2.3 ± 8.5%). This lower drop-out rate resulted in negative values for the excess drop-out rates. Contamination was present in 11 of 30 (37%) studies that reported on contamination.

The lowest contamination and drop-out rates were observed in studies with control groups that were offered an intervention after the intervention period (contamination in only 7.1% of studies, excess drop-out rate -4.7 ± 9.2%) (Table 2 and S3 Table) showing more detailed results for interventions during and after the intervention period).

**Table 2:** Contamination and drop-out rate by type of control group.

Type control group		N	Contamination^a^	Drop-out rate control group^b^(%)	Drop-out rate exercise group^b^(%)	Excess drop-out rate^b^ ^,^ ^c^(%)
A. Instruction control group BEFORE study intervention^d^	Exercise allowed	22	3/14 (21.4%)	10.2 ± 7.5	11.7 ± 7.1	-1.5 ± 6.9
	No change	18	8/16 (50.0%)	6.6 ± 6.6	9.8 ± 8.0	-3.2 ± 10.1
B. Intervention control group DURING study intervention^e^	Anything	21	6/15 (40.0%)	8.4 ± 7.9	12.5 ± 9.6	-4.1 ± 10.4
	None	19	5/15 (33.3%)	8.6 ± 6.7	9.2 ± 4.8	-0.6 ± 6.2
C. Intervention control group AFTER study intervention^f^	Anything	19	1/14 (7.1%)	5.8 ± 5.0	10.5 ± 7.9	-4.7 ± 9.2
	None	21	10/16 (62.5%)	11.2 ± 8.1	11.1 ± 7.3	-0.1 ± 7.1

^a^. For contamination results the data is given in number (percentages) of studies that reported contamination.

Contamination was reported in 30/40 (75%) studies.

^b^. For drop-out rates data are given in mean ± standard deviation of studies that reported drop-out. Drop- out rates were reported in 36/40 (90%) studies.

^c^. Excess drop-out rate = drop-out rate control group—drop-out rate exercise group.

^d^. Instruction ‘exercise allowed’ for control group included: exercise allowed during the study period, including no advice, asked or recommended to exercise

Instruction ‘no change’ for control group included: not to exercise, no change in exercise, continue exercise as it is.

^e^. Intervention for control group included: information about exercise, education (session) unrelated to exercises, keep exercise diary, use pedometers or accelerometers, phone calls unrelated to exercise, alternative intervention.

^f^. Study intervention was (partially) offered to control group after study intervention period, or information about exercise or exercise prescriptions were offered to control group after study intervention period.

Table 3, 4, and 5 show the results for contamination and drop-out for different combinations of the three design characteristics of the control group.

**Table 3:** Contamination and drop-out rate in the control group by (A) instruction before and (B) intervention during the study intervention period.

A. Instruction control group BEFORE study intervention^a^	B. Intervention control group DURING study period^b^	N	Contamination^c^	Drop-out rate control group^d^ (%)	Drop-out rate exercise group^d^(%)	Excess drop-out rate^d^ ^,^ ^e^(%)
Exercise allowed	Anything	11	1/7 (14.3%)	12.3 ± 9.3	13.1 ± 9.5	-0.8 ± 8.7
	None	11	2/7 (18.2%)	8.8 ± 5.8	10.7 ± 5.1	-1.9 ± 5.5
No change	Anything	10	5/8 (62.5%)	5.0 ± 4.5	12.0 ± 10.2	-7.1 ± 11.3
	None	8	3/8 (37.5%)	8.4 ± 8.3	7.2 ± 3.8	1.1 ± 7.0

^a^. Instruction ‘exercise allowed’ for control group included: exercise allowed during the study intervention period, including no advice, asked or recommended to exercise. Instruction ‘no change’ for control group included: not to exercise, no change in exercise, continue exercise as it is.

^b^. Intervention for control group included: information about exercise, education (session) unrelated to exercises, keep exercise diary, use pedometers or accelerometers, phone calls unrelated to exercise, alternative intervention.

^c^. For contamination data are given in number (percentages) of studies that reported contamination.

Contamination was reported in 30/40 (75%) studies.

^d^. For drop-out rates data are given in mean ± standard deviation of studies that reported drop out. Drop-out rates were reported in 36/40 (90%) studies.

^e^. Excess drop-out rate = drop-out rate control group—drop-out rate exercise group.

**Table 4:** Contamination and drop-out rate in the control group by (A) instruction before and (C) intervention after study intervention period.

A. Instruction control group BEFORE study intervention^a^	C. Intervention control group AFTER study period^b^	N	Contamination^c^	Drop-out rate control group^d^(%)	Drop-out rate exercise group^d^(%)	Excess drop-out rate^d^ ^,^ ^e^(%)
Exercise allowed	Anything	10	0/7 (0%)	7.1 ± 5.8	10.0 ± 5.4	-2.9 ± 6.1
	None	12	3/7 (42.9%)	13.8 ± 7.8	13.6 ± 8.5	0.2 ± 7.6
No change	Anything	9	1/7 (14.3%)	4.2 ± 3.7	11.2 ± 10.6	-7.0 ± 12.2
	None	9	7/9 (77.8%)	8.7 ± 8.0	8.5 ± 5.1	0.1 ± 7.0

^a^. Instruction ‘exercise allowed’ for control group included: exercise allowed during the study period, including no advice, asked or recommended to exercise. Instruction ‘no change’ for control group included: not to exercise, no change in exercise, continue exercise as it is.

^b^. Study intervention was (partially) offered to control group after study intervention period, or information about exercise or exercise prescriptions were offered to control group after study intervention period.

^c^. For contamination data are given in number (percentages) of studies that reported contamination.

Contamination was reported in 30/40 (75%) studies.

^d^. For drop-out rates data are given in mean ± standard deviation of studies that reported drop-out. Drop-out rates were reported in 36/40 (90%) studies.

^e^. Excess drop-out rate = drop-out rate control group—drop-out rate exercise group.

**Table 5:** Contamination and drop-out rate in the control group by (B) intervention during and (C) after study intervention period.

B. Intervention control group DURING study intervention^a^	C. Intervention control group AFTER study intervention^b^	N	Contamination^c^	Drop-out rate control group^d^(%)	Drop-out rate exercise group^d^(%)	Excess drop-out rate^d^ ^,^ ^e^(%)
Anything	Anything	7	0/5 (0%)	3.7 ± 4.3	13.7 ± 12.7	-10.0 ± 12.8
	None	14	6/10 (60.0%)	11.0 ± 8.4	11.9 ± 8.1	-0.9 ± 7.6
None	Anything	12	1/9 (11.1%)	6.9 ± 5.2	8.9 ± 4.0	-2.1 ± 5.9
	None	7	4/6 (66.7%)	11.5 ± 8.3	9.7 ± 6.2	1.8 ± 6.3

^a^. Intervention for control group included: information about exercise, education (session) unrelated to exercises, keep exercise diary, use pedometers or accelerometers, phone calls unrelated to exercise, alternative intervention.

^b^. Study intervention was (partially) offered to control group after study intervention period, or information about exercise or exercise prescriptions were offered to control group after study intervention period.

^c^. For contamination data are given in number (percentages) of studies that reported contamination.

Contamination was reported in 30/40 (75%) studies.

^d^. For drop-out rates data are given in mean ± standard deviation of studies that reported drop-out. Drop-out rates were reported in 36/40 (90%) studies.

^e^. Excess drop-out rate = drop-out rate control group—drop-out rate exercise group.

Table 3 shows the lowest contamination in control groups with an instruction that allowed exercise during the intervention period combined with any kind of intervention during the study (contamination in 14.3% of 7 studies). Lowest drop-out rates were present in control groups that used no change in exercise as an instruction combined with any kind of intervention during the study (excess drop-out -7.1 ± 11.3%).

Table 4 shows lowest contamination (0 out of 7 studies) in control groups with an instruction that allowed exercise combined with any kind of intervention after the study. Lowest drop-out rates (excess drop-out rate -7.0 ± 12.2%) were found in control groups that used no change in exercise as an instruction combined with any kind of intervention after the intervention period.

Table 5 shows least contamination (0 studies out of 5) and lowest excess drop-out rates (-10.0 ± 12.8%) in control groups that received an intervention both during and after the intervention period.

With regard to designs resulting in more contamination and higher drop-out rates, there was no combination that uniformly results in more contamination and higher drop-out rates. Highest excess drop-out rate (1.8 ± 6.3%) was present in control groups with no intervention during the intervention period in combination with no intervention after the intervention period; while highest contamination (77.8%) was in control groups with an instruction before the intervention period including ‘no change in exercise’ in combination with no intervention after the intervention period (Table 4–5).

In studies that included participants irrespective of their exercise history, mean excess drop-out rate was -1.3 ± 6.5 and contamination was 38.1%. In studies that only allowed inactive or unfit participants excess drop-out rate was -4.6 ± 11.9 and contamination was 33.3% (data not shown in Tables).

## Discussion

In this systematic review we present an overview of three different design characteristics of the control group and their influence on contamination and drop-out rates. Contamination was reported in 30/40 (75%) studies and drop-out rates were reported in 36/40 (90%) studies. Low contamination (7.1% of studies) and low drop-out rates (excess drop-out rate -4.7 ± 9.2) were found in control groups offered an intervention after the exercise intervention period, e.g. cross-over or exercise instruction. If control groups received an intervention both during (such as education sessions or regular telephone contacts) and after the intervention period, contamination (0%) and excess drop-out rates (-10.0 ± 12.8%) were even lower. In studies that included inactive or unfit patients only, excess drop-out rates were lower and contamination was slightly lower compared to studies without pre-trial exercise restrictions.

Contamination and drop-out rates are frequently mentioned as bias in control groups of exercise-oncology trials [4, 5, 43]. In order to prevent this bias, it is important to know which control group characteristics affect contamination and drop-out rates. In the literature little is known about the relationship between control group characteristics in exercise-oncology trials and contamination and drop-out rates. Waters et al. (2012) [7] investigated physical activity levels in control groups of physical intervention trials in the primary care setting. In line with our results, they found that minimal contamination was mostly found in control groups that were provided with some sort of intervention (defined as written and/or oral advice on physical activity).

In the present paper we focused on instructions or interventions for the control group as key factors that might influence contamination. In addition there are other possibilities in the design or analysis phase to handle contamination. Hertogh et al (2010) [6] providing recommendations about the expected behavior of the participants, not only at start but also throughout the study period. A better understanding of the study goals may result in better compliance [6]. Furthermore, in a pilot study we tested an adapted version of the Zelen design, in which only those who were randomized in the intervention group were informed about the exercise intervention, and for the patients randomized in the control group information about the detailed research aims was postponed until the end of the study [44]. However, patients were not enthusiastic about the incomplete information about the study, and the overall accrual into the study was low. Another option to account for contamination is in the data analyses phase. Researchers regularly perform per-protocol analyses when a high contamination rate exists. However, then random group allocation is not preserved and analyses can be highly biased because of selective non-compliance [45]. For example, control group participants who are highly motivated to exercise are more likely to initiate exercising [46]. Instrumental variable analysis is a promising method that could estimate, even in the presence of selective non-compliance, the causal effect of the intervention [6, 47].

A noteworthy finding of the current review is that drop-out rates were on average greater in the intervention group compared to drop-out in the control group. Future studies should focus on predictors of drop-out in both the intervention and the control group and also investigate whether these are distinct factors, which could be taken into account when designing exercise-oncology studies.

Although we performed a comprehensive systematic review, the following limitations have to be considered. The data indicate that the use of an alternative intervention in the control group during the study may reduce contamination and drop-out rates. However, whilst this might be the case, the effect of an alternative intervention upon the outcome of interest would also need to be considered to ensure that it does not mask a true effect of the experimental intervention. Additionally, difference in contamination or drop-out rates might not only be based on different instructions or interventions offered to the control group. Other factors, such as the type of cancer, pre-trial exercise restriction, sample size, age of participants, treatments received or length of intervention [6] might influence contamination and drop-out rates. Unfortunately, because of limited number of studies we are not able to perform inferential statistical analyses to control for confounding factors. We were also not able to statistically test differences between groups. Furthermore, for the same reason, we were not able to make the categories of control groups more precise. For example, the category ‘any’ intervention during the intervention period included both information about exercise and an alternative intervention like stretching, while differences in terms of contamination and drop out between these subcategories might exist. Even the current percentages that we present are based on a limited numbers of studies (often below 10) and thus should be interpreted with caution. Additionally, it is possible that studies with mainly low contamination were published and therefore publication bias might be an issue. Finally, in 25% of the included studies contamination was not reported. We recommend authors from exercise-oncology trials to assess physical activity levels in the control group and report contamination for a careful interpretation of the results.

## Conclusion

The results from this systematic review suggest that control groups receiving an intervention after the exercise intervention period have low contamination and low drop-out rates. If control groups receive an additional intervention during the intervention period, this effect seems even stronger. For future exercise oncology trials, it might be beneficial to offer an intervention to the control group both during and after the study period to prevent contamination and drop out. The most optimal type of control group intervention needs further investigation.

## Supporting Information

S1 Table Detailed literature search. (DOCX)

S2 Table Characteristics of each individual study included in the review. Abbreviations: Ref—references; NR—not reported
a. Instruction control group: BEFORE study intervention period: (A) not to exercise/change exercise/ continue as is (all implying no change); (B) allowed to exercise such as no advice, do what they want; (C) asked/ recommended to exercise
b. Intervention control group DURING study intervention period: (A) information about exercise; (B) education (session) unrelated to exercises; (C) phone calls unrelated to exercise; (D) stretching program; (E) relaxation training; (G) psycho-education; (H) keep exercise diary, regular PA questionnaire; (I) use pedometers/accelerometers; (J) outcome assessment during intervention
c. Intervention control group AFTER study intervention period: A) full cross-over; (B) partial cross-over;(C) single session;(D) exercise prescription; (E) Information about exercise
d. Control patients received weekly telephone calls by project director to report level of exercise and answer questions and provide equal contact and attention
e. +I, only 1st 2 weeks and last week
f. + access to the full range of psychosocial services
g. + light-intensity resistance exercise (not individualized)
h. 2-page leaflet "Exercise after cancer diagnosis"
i. Support to construct own personalized exercise plan and invited to join GP exercise referral scheme
j. Not discouraged from performing normal activities, but advised to rest and thinks easy if they became fatigue
* We rated contamination (yes or no) for each study as defined by Waters et al. (2012) (44) as an increase of ≥ 60 minutes (4 MET hours) of moderate to vigorous physical activity per week in the control group, or a 10% increase in the proportion of participants meeting the study exercise prescription (e.g. physical activity guidelines). In addition, contamination was also scored to be present if reported by the authors using slightly different definitions. For example, in several home-based exercise trials contamination was defined as exercising (moderate/strenuous) >60 minutes per week (32).
(DOCX)

S3 Table Contamination and excess drop-out rates by detailed interventions during and after the study intervention period a. For contamination results data is given in number (percentages) of studies that reported contamination. Contamination was reported in 30/40 (75%) studies.
b. For drop-out rates data is given in mean ± standard deviation of studies that reported drop-out. Drop-out rates were reported in 36/40 (90%) studies.
c. Excess drop-out rate = drop-out rate control group—drop-out rate exercise group.
(DOCX)
