# Supplementary material for: Control Group Design, Contamination and Drop-Out in Exercise Oncology Trials: A Systematic Review
Source: PLoS One. 2015 Mar 27;10(3):e0120996. doi: 10.1371/journal.pone.0120996 (PMC4376879; doi:10.1371/journal.pone.0120996)
Supplement: S1 Table — (DOCX) [file pone.0120996.s001.docx]

**S1 Table. Detailed literature search**

**Search Medline**

((((((((Exercise[Title/Abstract]) OR Exercize[Title/Abstract]) OR Training[Title/Abstract]) OR Walking[Title/Abstract]) OR Physical[Title/Abstract]) OR Sport[Title/Abstract])) AND ((((((((trial[Title/Abstract]) OR trials[Title/Abstract]) OR randomized[Title/Abstract]) OR randomize[Title/Abstract]) OR randomised[Title/Abstract]) OR randomise[Title/Abstract]) OR RCT[Title/Abstract]) OR RCTs[Title/Abstract])) AND ((((((((((((((((((Cancer[Title/Abstract]) OR Cancers[Title/Abstract]) OR Neoplasm[Title/Abstract]) OR Neoplasms[Title/Abstract]) OR leukemia[Title/Abstract]) OR leukemi[Title/Abstract]) OR tumor[Title/Abstract]) OR tumour[Title/Abstract]) OR malignancy[Title/Abstract]) OR malignancies[Title/Abstract]) OR carcinoma[Title/Abstract]) OR carcinomas[Title/Abstract]) OR adenocarcinoma[Title/Abstract]) OR adenocarcinomas[Title/Abstract]) OR lymphoma[Title/Abstract]) OR lymphomas[Title/Abstract]) OR chemotherapy[Title/Abstract]) OR chemo[Title/Abstract])

**Search Embase**

exercise:ab,ti OR exercize:ab,ti OR training:ab,ti OR walking:ab,ti OR physical:ab,ti OR sport;ab,ti AND (trial:ab,ti OR trials:ab,ti OR randomized:ab,ti OR randomize:ab,ti OR randomised:ab,ti OR randomise:ab,ti OR rct:ab,ti OR rcts;ab,ti) AND (cancer:ab,ti OR cancers:ab,ti OR neoplasm:ab,ti OR neoplasms:ab,ti OR leukemia:ab,ti OR leukemi:ab,ti OR tumor:ab,ti OR tumour:ab,ti OR malignancy:ab,ti OR malignancies:ab,ti OR carcinoma:ab,ti OR carcinomas:ab,ti OR adenocarcinoma:ab,ti OR adenocarcinomas:ab,ti OR lymphoma:ab,ti OR lymphomas:ab,ti OR chemotherapy:ab,ti OR chemo;ab,ti) AND [embase]/lim AND [2012-2014]/py AND ('clinical trial'/de OR 'clinical trial (topic)'/de OR 'randomized controlled trial'/de OR 'randomized controlled trial (topic)'/de)

**Search Cinahl**

Exercise AND cancer
