# Supplementary material for: Control Group Design, Contamination and Drop-Out in Exercise Oncology Trials: A Systematic Review
Source: PLoS One. 2015 Mar 27;10(3):e0120996. doi: 10.1371/journal.pone.0120996 (PMC4376879; doi:10.1371/journal.pone.0120996)
Supplement: S2 Table — Abbreviations: Ref—references; NR—not reported a. Instruction control group: BEFORE study intervention period: (A) not to exercise/change exercise/ continue as is (all implying no change); (B) allowed to exercise such as no advice, do what they want; (C) asked/ recommended to exercise b. Intervention control group DURING study intervention period: (A) information about exercise; (B) education (session) unrelated to exercises; (C) phone calls unrelated to exercise; (D) stretching program; (E) relaxation training; (G) psycho-education; (H) keep exercise diary, regular PA questionnaire; (I) use pedometers/accelerometers; (J) outcome assessment during intervention c. Intervention control group AFTER study intervention period: A) full cross-over; (B) partial cross-over;(C) single session;(D) exercise prescription; (E) Information about exercise d. Control patients received weekly telephone calls by project director to report level of exercise and answer questions and provide equal contact and attention e. +I, only 1st 2 weeks and last week f. + access to the full range of psychosocial services g. + light-intensity resistance exercise (not individualized) h. 2-page leaflet "Exercise after cancer diagnosis" i. Support to construct own personalized exercise plan and invited to join GP exercise referral scheme j. Not discouraged from performing normal activities, but advised to rest and thinks easy if they became fatigue * We rated contamination (yes or no) for each study as defined by Waters et al. (2012) (44) as an increase of ≥ 60 minutes (4 MET hours) of moderate to vigorous physical activity per week in the control group, or a 10% increase in the proportion of participants meeting the study exercise prescription (e.g. physical activity guidelines). In addition, contamination was also scored to be present if reported by the authors using slightly different definitions. For example, in several home-based exercise trials contamination was defined as exercising (moderate/st [file pone.0120996.s002.docx]

**S2 Table. Characteristics of each individual study included in the review**

Abbreviations: Ref – references; NR – not reported

a. Instruction control group: BEFORE study intervention period: (A) not to exercise/change exercise/ continue as is (all implying no change); (B) allowed to exercise such as no advice, do what they want; (C) asked/ recommended to exercise

b. Intervention control group DURING study intervention period: (A) information about exercise; (B) education (session) unrelated to exercises; (C) phone calls unrelated to exercise; (D) stretching program; (E) relaxation training; (G) psycho-education; (H) keep exercise diary, regular PA questionnaire; (I) use pedometers/accelerometers; (J) outcome assessment during intervention

c. Intervention control group AFTER study intervention period: A) full cross-over; (B) partial cross-over;(C) single session;(D) exercise prescription; (E) Information about exercise

d. Control patients received weekly telephone calls by project director to report level of exercise and answer questions and provide equal contact and attention

e. +I, only 1st 2 weeks and last week

f. + access to the full range of psychosocial services

g. + light-intensity resistance exercise (not individualized)

h. 2-page leaflet "Exercise after cancer diagnosis"

i. Support to construct own personalized exercise plan and invited to join GP exercise referral scheme

j. Not discouraged from performing normal activities, but advised to rest and thinks easy if they became fatigued

* We rated contamination (yes or no) for each study as defined by Waters et al. (2012) (44) as an increase of ≥ 60 minutes (4 MET hours) of moderate to vigorous physical activity per week in the control group, or a 10% increase in the proportion of participants meeting the study exercise prescription (e.g. physical activity guidelines). In addition, contamination was also scored to be present if reported by the authors using slightly different definitions. For example, in several home-based exercise trials contamination was defined as exercising (moderate/strenuous) >60 minutes per week (32).

| Ref | Instruction control group: BEFORE study intervention period (a) | Intervention control group  DURING study intervention period (b) | Intervention control group AFTER intervention study period (c) | Total study participants | Cancer type | Timing exercise program | Setting exercise program | Type exercise program | Mean age (years) | Contamination * | Remark | Dropout rate control group  (%) | Dropout rate exercise group (%) | Excess dropout rate  (control – exercise group) (%) |
| --- | --- | --- | --- | --- | --- | --- | --- | --- | --- | --- | --- | --- | --- | --- |
| 9 | B | None | A | 269 | Mixed | During chemo | Supervised | Combi- nation | 47 | No | Increase of < 60 minutes (4 MET hours) of moderate to vigorous physical activity per week in control group | 12.7 | 12.6 | 0.1 |
| 10 | B | C | None | 53 | Lung | Other | Both | Combi- nation | 64 | NR |  | 19.2 | 14.8 | 4.4 |
| 11 | A | None | D | 43 | Mixed | After treatment | Both | Aerobic | 51 | No | Increase of < 60 minutes (4 MET hours) of moderate to vigorous physical activity per week in control group | 5.0 | 8.7 | -3.7 |
| 12 | B | None | D | 75 | Breast | After treatment | Supervised | Aerobic | 56 | No | Increase of < 60 minutes (4 MET hours) of moderate to vigorous physical activity per week in control group | 13.2 | 8.1 | -5.0 |
| 12 | B | None | D | 50 | Breast | Mixed | Home based | Aerobic | 54 | NR |  | 8.0 | 12.0 | -4.0 |
| 4 | A | C+H (d) | D | 102 | Colo-rectal | After treatment | Home based | Aerobic | 60 | Yes | Increase of > 10% in the proportion of participants meeting the study exercise prescription in the control group | 6.1 | 10.1 | -4.1 |
| 13 | B | None | A | 53 | Breast | After treatment | Supervised | Aerobic | 59 | No | Increase of < 60 minutes (4 MET hours) of moderate to vigorous physical activity per week in control group | 0 | 4.0 | -4.0 |
| 14 | A | H | D | 108 | Mixed | After treatment | Home based | Aerobic | 52 | No | Increase of < 60 minutes (4 MET hours) of moderate to vigorous physical activity per week in control group | 6.3 | 15.0 | -8.8 |
| 7 | A | None | B | 122 | Lymphoma | Mixed | Supervised | Aerobic | 53 | No | Increase of < 10% in the proportion of participants meeting the study exercise prescription in the control group | 3.2 | 5.0 | -1.8 |
| 15 | A | None | B | 242 | Breast | During chemo | Supervised | Combi- nation | 49 | No | Increase of < 10% in the proportion of participants meeting the study exercise prescription in the control group | 11.0 | 5.6 | 5.4 |
| 16 | A | None | None | 55 | Mixed | Other | Supervised | Aerobic | 56 | No | Increase of < 60 minutes (4 MET hours) of moderate to vigorous physical activity per week in control group | 0 | 3.8 | -3.8 |
| 18 | A | None | None | 108 | Breast | After treatment | Supervised | Aerobic | 51 | No | Increase of <10% in the proportion of participants meeting the study exercise prescription in the control group | 13.2 | 2.9 | 10.2 |
| 18 | A | D | None | 108 | Mixed | After treatment | Supervised | Aerobic | 51 | Yes | Increase of >10% in the proportion of participants meeting the study exercise prescription in the control group | 0 | 2.9 | -2.9 |
| 19 | B | E | None | 72 | Mixed | Mixed | Supervised | Combi- nation | 58 | NR |  | NR | NR | NR |
| 20 | B | None | A | 119 | Mixed | During + after chemo | Home based | Aerobic | 51 | No | Increase of < 10% in the proportion of participants meeting the study exercise prescription in the control group | 2.8 | 11.4 | -8.6 |
| 20 | A | C | None | 119 | Mixed | During + after chemo | Home based | Aerobic | 51 | Yes | Increase of > 10% in the proportion of participants meeting the study exercise prescription in the control group | 2.6 | 11.4 | -8.8 |
| 21 | B | B | None | 60 | Breast | After treatment | Both | Combi- nation | 52 | NR |  | 0 | 5.0 | -5.0 |
| 22 | B | None | A | 57 | Prostate | Other | Supervised | Combi- nation | 70 | No | Increase of < 60 minutes (4 MET hours) of moderate to vigorous physical activity per week in control group | 3.6 | 3.4 | 0.1 |
| 23 | C | A+I | None | 100 | Prostate | After treatment | Both | Combi- nation | 72 | No | Increase of < 60 minutes (4 MET hours) of moderate to vigorous physical activity per week in control group | 16.0 | 28.0 | -12.0 |
| 24 | A | C + H (e) | None | 126 | Mixed | Mixed | Home based | Aerobic | 60 | Yes | Increase of > 10% in the proportion of participants meeting the study exercise prescription in the control group | 10.8 | 6.8 | 3.9 |
| 25 | B | None | None | 194 | Breast | Other | Supervised | Combi- nation | 52 | Yes | Increase of > 60 minutes (4 MET hours) of moderate to vigorous physical activity per week in control group | 6.7 | 7.5 | -0.8 |
| 26 | B | B (f) | None | 268 | Mixed | During chemo | Home based | Aerobic | 58 | No | Increase of < 60 minutes (4 MET hours) of moderate to vigorous physical activity per week in control group | NR | NR | NR |
| 26 | B | G | None | 268 | Mixed | During chemo | Home based | Aerobic | 58 | No | Increase of < 60 minutes (4 MET hours) of moderate to vigorous physical activity per week in control group | NR | NR | NR |
| 27 | NR | NR | NR | 251 | Breast | After treatment | Supervised | Combi- nation | 52 | Yes | Increase of > 10% in the proportion of participants meeting the study exercise prescription in the control group | 11.6 | 11.0 | 0.5 |
| 28 | B | B | None | 45 | Colo-rectal | During chemo | Supervised | Combi- nation | 57 | No | Increase of < 60 minutes (4 MET hours) of moderate to vigorous physical activity per week in control group | 20.8 | 4.8 | 16.1 |
| 29 | B | D (g) | None | 52 | Head & Neck | After treatment | Supervised | Resistance | 52 | NR |  | 8.0 | 7.4 | 0.6 |
| 30 | A | C | A | 58 | Breast | After treatment | Supervised | Combi- nation | 55 | NR |  | 0 | 0 | 0 |
| 8 | A | C | None | 119 | Breast | Mixed | Home based | Aerobic | 52 | Yes | Increase of > 10% in the proportion of participants meeting the study exercise prescription in the control group | 8.5 | 10.0 | -1.5 |
| 31 | B | A (h) | D (i) | 203 | Breast | During+ after chemo | Supervised | Combi- nation | 52 | No | Increase of < 60 minutes (4 MET hours) of moderate to vigorous physical activity per week in control group | 9.8 | 18.8 | -9.0 |
| 32 | A | None | None | 573 | Breast | After treatment | Both | Combi- nation | 52 | Yes | Increase of > 60 minutes (4 MET hours) of moderate to vigorous physical activity per week in control group | 6.3 | 7.4 | -1.1 |
| 33 | B | None | D | 42 | Head & Neck | During chemo-radiation | Supervised | Combi- nation | 52 | NR |  | 4.2 | 16.7 | -12.5 |
| 36 | A | None | A | 121 | Prostate | Other | Supervised | Combi- nation | 66 | Yes | Increase of > 10% in the proportion of participants meeting the study exercise prescription in the control group | 2.4 | 10.0 | -7.6 |
| 35 | B | None | A | 155 | Prostate | Other | Supervised | Resistance | 68 | NR |  | 16.4 | 9.8 | 6.7 |
| 34 | C | None | None | 123 | Breast | During+ after chemo | Both | Aerobic | 51 | NR |  | 17.1 | 20.6 | -3.6 |
| 37 | A | H | D | 71 | Mixed | During+ after chemo | Home based | Aerobic | 52 | NR |  | 0 | 35.1 | -35.1 |
| 38 | A | None | None | 111 | Mixed | After treatment | Home based | Aerobic | 39 | Yes | Increase of > 60 minutes (4 MET hours) of moderate to vigorous physical activity per week in control group | 25.7 | 14.5 | 11.2 |
| 39 | A | C | None | 62 | Breast | During chemo | Home | Aerobic | 50 | Yes | Increase of > 60 minutes (4 MET hours) of moderate to vigorous physical activity per week in control group | 10.8 | 17.1 | -6.3 |
| 40 | B (j) | C+H | E | 66 | Prostate | Other | Home | Aerobic | 69 | No | Increase of < 60 minutes (4 MET hours) of moderate to vigorous physical activity per week in control group | 0 | 3.0 | -3.0 |
| 41 | B | C | None | 105 | Mixed | Other | Both | Combi- nation | 49 | Yes | Increase of > 60 minutes (4 MET hours) of moderate to vigorous physical activity per week in control group | 24.5 | 23.1 | 1.4 |
| 42 | A | C | D | 40 | Breast | During  chemo | Home | Aerobic | 52 | No | Increase of < 60 minutes (4 MET hours) of moderate to vigorous physical activity per week in control group | NR | NR | NR |
