# Supplementary material for: Control Group Design, Contamination and Drop-Out in Exercise Oncology Trials: A Systematic Review
Source: PLoS One. 2015 Mar 27;10(3):e0120996. doi: 10.1371/journal.pone.0120996 (PMC4376879; doi:10.1371/journal.pone.0120996)
Supplement: S3 Table — a. For contamination results data is given in number (percentages) of studies that reported contamination. Contamination was reported in 30/40 (75%) studies. b. For drop-out rates data is given in mean ± standard deviation of studies that reported drop-out. Drop-out rates were reported in 36/40 (90%) studies. c. Excess drop-out rate = drop-out rate control group—drop-out rate exercise group. (DOCX) [file pone.0120996.s003.docx]

**S3 Table. Contamination and excess drop-out rates by detailed interventions during and after the study intervention period**

a. For contamination results data is given in number (percentages) of studies that reported contamination.

Contamination was reported in 30/40 (75%) studies.

b. For drop-out rates data is given in mean ± standard deviation of studies that reported drop-out. Drop-out rates were reported in 36/40 (90%) studies.

c. Excess drop-out rate = drop-out rate control group – drop-out rate exercise group.

| Type control group | | N | Contamination^a^ | Excess drop-out rate^b,c^ (%) |
| --- | --- | --- | --- | --- |
| B. Intervention control group DURING study intervention period | Information about exercise/education (session) unrelated to exercises / keep exercise diary/use pedometers/ accelerometers | 7 | 0/5 (0%) | -9.0±16.3 |
|  | Phone calls unrelated to exercise (with or without exercise screening) | 10 | 5/8 (62.5%) | -1.6±4.9 |
|  | Alternative intervention | 4 | 1/1 (50%) | -1.1±2.5 |
|  | None | 19 | 5/15 (33.3%) | -0.6±6.2 |
| C. Intervention control group AFTER study intervention period | Full or partial cross-over | 9 | 1/7 (14.3%) | -1.1±5.2 |
|  | Information about exercise/exercise prescription | 10 | 0/7 (0%) | -8.4±11.2 |
|  | None | 21 | 10/16 (62.5%) | 0.4±7.1 |
